# Estimating the proportion of Medicaid-eligible pregnant women in Louisiana who do not get abortions when Medicaid does not cover abortion

**DOI:** 10.1186/s12905-019-0775-5

**Published:** 2019-06-19

**Authors:** Sarah C. M. Roberts, Nicole E. Johns, Valerie Williams, Erin Wingo, Ushma D. Upadhyay

**Affiliations:** 1 Advancing New Standards in Reproductive Health (ANSIRH), Department of Obstetrics, Gynecology, and Reproductive Sciences, University of California, San Francisco, 1330 Broadway, Suite 1100, Oakland, CA 94612 USA; 2 Department of Obstetrics and Gynecology, Louisiana State University School of Medicine, 3700 St. Charles Avenue, 5th floor, New Orleans, LA 70115 USA; 3 Present address: Center on Gender Equity and Health, University of California, San Diego, 9500 Gilman Dr, La Jolla, CA 92093 USA

**Keywords:** Abortion, Medicaid, Policy, Pregnancy, women’s health, Barriers to care

## Abstract

**Background:**

To estimate the proportion of pregnant women in Louisiana who do not obtain abortions because Medicaid does not cover abortion.

**Methods:**

Two hundred sixty nine women presenting at first prenatal visits in Southern Louisiana, 2015–2017, completed self-administered iPad surveys and structured interviews. Women reporting having considered abortion were asked whether Medicaid not paying for abortion was a reason they had not had an abortion. Using study data and published estimates of births, abortions, and Medicaid-covered births in Louisiana, we projected the proportion of Medicaid births that would instead be abortions if Medicaid covered abortion in Louisiana.

**Results:**

28% considered abortion. Among women with Medicaid, 7.2% [95% CI 4.1–12.3] reported Medicaid not paying as a reason they did not have an abortion. Existing estimates suggest 10% of Louisiana pregnancies end in abortion. If Medicaid covered abortion, this would increase to 14% [95% CI 12, 16]. 29% [95% CI 19, 41] of Medicaid eligible pregnant women who would have an abortion with Medicaid coverage, instead give birth.

**Conclusions:**

For a substantial proportion of pregnant women in Louisiana, the lack of Medicaid funding remains an insurmountable barrier to obtaining an abortion. Forty years after the Hyde Amendment was passed, lack of Medicaid funding for abortion continues to have substantial impacts on women’s ability to obtain abortions.

## Background

The Hyde Amendment, which restricts use of federal Medicaid dollars to pay for abortion, is one of the longest running abortion restrictions [1]. Seventeen states use state funding to pay for abortion for Medicaid eligible women [2], meaning that in most U.S. states, there is no public funding to pay for abortion for low-income women. Even in the midst of hundreds of new restrictive abortion policies enacted between 2011 and 2017 [3], policy discussions continue to focus on Medicaid coverage for abortion [1, 2, 4, 5].

Lack of Medicaid funding impacts the three-fourths of women obtaining abortions in the U.S. who are of low-income [6]. Out-of-pocket costs for abortion are over one-third of monthly personal income for about half of abortion patients [7]. Having to pay out of pocket has financial implications for women, including lost wages and delay in paying bills [8].

Research about impacts of the Hyde Amendment has been conducted for almost as long as the policy has been in effect. Prior to 2009, most research focused on the extent to which restricting Medicaid funding for abortion affected women’s ability to obtain abortions [9]. A systematic review of that body of literature noted methodological flaws, but concluded that about one-fourth (18–37%) of women who would have had Medicaid-covered abortions instead gave birth when funding was unavailable [9]. A small number of studies examined whether restricting Medicaid funding for abortion led to delays in obtaining abortions and impacted other outcomes – such as complications from illegal abortions and birth outcomes [9].

Since 2009, researchers have continued to study impacts of restricted Medicaid funding for abortion. Methodologically sophisticated studies have documented Medicaid funding restrictions’ impact on maternal morbidity and infant mortality [10, 11]. Other research examined women’s and provider’s experiences with Medicaid coverage and found that, even when Medicaid can pay for abortion, it sometimes does not, leading to delays and financial and emotional impacts on women obtaining abortions [8, 12–15].

Recent literature has not estimated the impact of lack of Medicaid coverage for abortion. While the systematic review that produced the one-fourth estimate of those who would have had a Medicaid covered abortion if coverage was available was published in 2009, much of the research behind that estimate was published in the 1980s and 1990s [9]. A key question is whether this estimate is still relevant. Another key unanswered question is what are characteristics of women who do not obtain abortions when Medicaid restrictions are in effect?

## Methods

### Study design

The Louisiana Abortion Prenatal Study was designed to study impacts of Louisiana’s abortion restrictions [16]. We recruited participants at three university-affiliated prenatal care facilities in Southern Louisiana that serve pregnant women who have or are eligible for Medicaid. We describe the study methods in detail elsewhere [17]. Briefly, between June 2015 and May 2017, we recruited women at their first prenatal care visit. Participants first completed self-administered iPad surveys; they then completed in-clinic structured interviews with a research coordinator. The Institutional Review Boards at the University of California, San Francisco and The Louisiana State University Health Sciences Campus granted ethical approval for this study.

In this manuscript, we aim to estimate the proportion of women who give birth instead of have an abortion because neither federal Medicaid nor state funds covers abortion for low-income women in Louisiana. We chose Louisiana because Louisiana state Medicaid does not cover abortion [2]. Abortion funds are a set of private organizations that seek to address limitations in insurance coverage and geographic access to abortion [18]. To help pay for the costs of a low-income woman’s abortion, these abortion funds provide subsidies to health care facilities to cover some or all of the costs of the abortion. Some funds are large (covering thousands of abortions per year) and others are small (covering only a few abortions per year) [19]. The local abortion fund in Southern Louisiana only covers a small portion of costs at the abortion clinics in Southern Louisiana. At the national level, the price for an abortion is more than $500 and the adjusted prices are higher in states that have more restrictive abortion policies, such as Louisiana [20]. Average out-of-pocket costs for abortion (including abortion funds or clinic discounts) is more than $300 for first trimester and close to $600 across all gestations [7]. Women in states where abortion for low-income women is covered by state funds pay, on average, $0 out of pocket [7].

At the time we began the study in 2015, Louisiana had five abortion clinics [21], three in the southern part of the state. By the time we finished recruitment in 2017, Louisiana had three abortion clinics, with two in the southern part of the state. Neither the prenatal care clinics where we recruited nor the local Planned Parenthood facilities provide abortions.

### Study procedures

In each recruitment facility, a research coordinator approached all women over 18 who presented for their first prenatal care appointment during the study time period and who spoke English. During the first year of recruitment, we began recruiting Spanish-speaking women. Women who were ineligible included those who were under 18, not pregnant, receiving a noninitial prenatal visit, not English or Spanish speaking, or incarcerated. As reported previously, of eligible individuals, 86% consented to participate [17].

Research coordinators first obtained informed consent. They then instructed participants on how to complete self-administered iPad surveys and left them to complete surveys independently. After participants completed iPad surveys, the research coordinator conducted brief in-clinic structured interviews with participants.

### Measures

The primary outcome was whether Medicaid not paying for abortion was a reason a pregnant woman had not had an abortion. To assess this outcome, we asked multiple questions. As a first step towards assessing whether Medicaid was a reason for not having an abortion, the iPad survey asked, “Have you considered abortion for this pregnancy even for just one second?” In the in-clinic interview, the research coordinator repeated this question verbatim. As described previously, reporting having considered abortion for this pregnancy was consistent across modes; 94% of participants reported consistently across modes [17]. To assess the main outcome, in in-clinic interviews, participants who reported considering abortion in the in-clinic interviews were asked: “Medicaid in Louisiana does not pay for abortion. Was Medicaid not paying for abortion part of why you have not had an abortion?” Those who responded yes were considered to have not had an abortion because Medicaid did not cover it.

As a secondary measure of the outcome, we used data from open-ended responses to questions about reason(s) for not having an abortion and the main reason for not having an abortion. In the in-clinic interview, the research coordinator asked participants who reported they had considered abortion “even for just one second” a series of questions on concrete actions they may have taken to seek an abortion. Specifically, the research coordinator asked about the following concrete actions, whether they had: called an abortion clinic, made an appointment for an abortion, and went to the state-mandated abortion counseling visit and the abortion appointment. Once a participant responded that she had not taken the next concrete action in the series of possible actions, the research coordinator asked an open-ended question about her reason(s) for not having taken that step and then asked her to specify her main reason for not having an abortion. We trained research coordinators to: document responses verbatim, use neutral probes for clarity, and obtain more detail from participants. We classified responses that included “fund”, “money”, “price”, “insurance”, “dollars”, “$”, “cost” as financial reasons for not having an abortion. We did face validity checks to ensure responses were related to financial reasons.

We used additional variables as validity checks for reporting Medicaid as a reason for not having an abortion. We asked which pregnancy outcome women preferred upon pregnancy discovery and which pregnancy outcome they preferred now (upon prenatal care entry). In the iPad survey, we asked: “Please think back to the week right after you found out you were pregnant. Please tell me which option you preferred the **week right after** you found out you were pregnant. Having the baby; Adoption or having someone else raise it; Having an abortion.” Then, with the same answer options, we asked, “Next, please tell us which option you prefer **now**.” We assessed pregnancy planning using the London Measure of Unplanned Pregnancy; for ease of interpretation, we categorized the scale as unplanned, ambivalent, or planned [22]. We measured decisional certainty using the Decisional Conflict Scale, a 16-item scale used in multiple areas of health care to measure people’s certainty around different health care decisions; for ease of interpretation, we categorized the scale as high certainty, medium certainty, and low certainty [23].

To assess whether participants who reported Medicaid not paying as a reason for not having an abortion may have proceeded to have an abortion after the interview, we asked “Are you still considering having an abortion?”, after the open-ended questions about reasons for not having an abortion.

As people sometimes report more than one reason for not having an abortion after considering one [17], we used responses to the open-ended questions about reasons for not having an abortion that we previously coded into personal reason, interpersonal reason, healthcare/other organization interaction, and policy-related reason. Specifically, responses coded as policy-related reasons were used as a check on Medicaid-related reason. Responses could fall into more than one category.

We assessed characteristics, including age (continuous), race/ethnicity (categorical), parity (categorical), education (categorical), employment (dichotomous), public assistance receipt (dichotomous), food insecurity (dichotomous), housing insecurity (dichotomous), insurance status (categorical), relationship with man involved in the pregnancy (categorical), past-year alcohol use disorder risk (dichotomous from AUDIT-C scale, number of drinks modified from 6 to 4 [24]), past-year drug use (dichotomous), and past-year tobacco use (dichotomous).

#### Births and abortions

We used published estimates of the number of births and abortions in Louisiana in 2015 as well as guidance on estimating the number of miscarriages based on birth and abortion data [25–27] to estimate the number of abortions, births, and miscarriages in Louisiana in 2015.We obtained published estimates of the proportion of Louisiana births paid for by Medicaid in 2015 [28].

### Analysis

We estimated the proportion of participants who reported that they did not have an abortion because Medicaid did not pay, including 95% Confidence Intervals (CIs). We assessed whether this estimate varied if we instead used coded responses from open-ended questions. We then estimated this proportion among women with Medicaid insurance, including 95% CIs.

For validity checks, we examined associations between Medicaid not paying as a reason and pregnancy outcome preference at pregnancy discovery, pregnancy outcome preference at prenatal care entry, pregnancy planning, and decisional certainty using chi-square tests and Fisher’s exact tests.

We then estimated the proportion of women who gave birth instead of having an abortion due to Medicaid not covering abortion. We used data on the number of abortions and births to Louisiana residents in 2015 as well as guidance on estimating the number of miscarriages based on abortions and births to estimate total number of Louisiana births, miscarriages, and abortions in 2015. We used published estimates of births paid for by Louisiana Medicaid and study estimates of the proportion with Medicaid insurance who reported not having an abortion because Medicaid would not pay to estimate the number of births paid for by Medicaid that would instead be abortions if Medicaid covered abortion. We added this number to published estimates of abortions to estimate projected number of abortions in Louisiana if Medicaid covered abortion. We then calculated proportion of women who give birth instead of having an abortion because Medicaid does not cover it through the equation (Projected abortions – Actual abortions)/Projected abortions. We repeated these steps, replacing estimates of proportions of women who reported that they did not have an abortion because Medicaid did not pay with lower and upper bounds of our estimate of the proportion who reported not having an abortion due to Medicaid not paying, to get a 95% CI.

We then described characteristics of women who report not having an abortion because Medicaid did not pay. We conducted bivariate analyses using t-tests for continuous and chi-square tests or Fisher’s exact tests for dichotomous and categorical variables to identify characteristics associated with not having an abortion because Medicaid did not pay among those who had Medicaid insurance.

## Results

### Sample

Two hundred eighty five participants consented to participate. 269 completed structured interviews and 265 responded to the question about whether lack of Medicaid coverage for abortion was a reason for not having an abortion. Having considered abortion was not associated with interview completion [17]. Study population characteristics are in Table 1. Most participants were Black (72%), low socio-economic status (65%), received public assistance in the past year; 50% were food insecure, 33% housing insecure, and 65% had given birth previously. About one-fourth reported past-year alcohol use disorder risk, 16% past-year drug use, and 28% past-year tobacco use. Few pregnancies were planned (25%), more than ten percent preferred abortion upon pregnancy discovery (14%) and most were certain of their decision to continue pregnancy by the time they entered prenatal care (98%). [See Table 1].

**Table 1:** Sample characteristics and characteristics associated with reporting Medicaid as a reason for not having an abortion

	Total % (N)	Medicaid population % (N)	Medicaid as a reason (Medicaid population denominator) % (N)		*P*-value among Medicaid population
			Yes	No	
N	265	167	12	155	
Age					0.50
Age (mean)	27	27	26	27	
Race/ethnicity					0.87
White	8% (22)	11% (19)	8% (1)	12% (18)	
Black	72% (190)	74% (124)	75% (9)	74% (115)	
Hispanic/Latina	15% (39)	10% (16)	8% (1)	10% (15)	
Other/Multi	5% (14)	5% (8)	8% (1)	4% (7)	
Parity					0.51
0	35% (94)	33% (55)	17% (2)	34% (53)	
1	26% (68)	25% (41)	33% (4)	24% (37)	
2 or more	39% (103)	42% (71)	50% (6)	42% (65)	
Education					0.03
Less than HS	25% (65)	23% (39)	42% (5)	22% (34)	
HS or GED	48% (127)	48% (80)	58% (7)	47% (73)	
Some or completed College	28% (73)	29% (48)	0% (0)	31% (48)	
Currently employed					0.23
No	52% (137)	55% (91)	75% (9)	53% (82)	
Yes	48% (126)	45% (75)	25% (3)	47% (72)	
Public assistance					0.52
No	35% (92)	26% (44)	33% (4)	26% (40)	
Yes	65% (169)	74% (123)	67% (8)	74% (115)	
Food insecure					0.07
No	50% (133)	52% (87)	25% (3)	54% (84)	
Yes	50% (131)	48% (80)	75% (9)	46% (71)	
Housing insecure					0.20
No	67% (176)	68% (114)	50% (6)	70% (108)	
Yes	33% (88)	32% (53)	50% (6)	30% (47)	
Relationship					0.003
Husband/fiancé	29% (76)	26% (44)	0% (0)	28% (44)	
Boyfriend/partner	51% (135)	53% (88)	42% (5)	54% (83)	
Ex/friend/none/don’t know	20% (53)	21% (35)	58% (7)	18% (28)	
Alcohol use disorder risk					< 0.001
No	76% (200)	75% (125)	25% (3)	79% (122)	
Yes	24% (64)	25% (42)	75% (9)	21% (33)	
Drug use					0.03
No	84% (219)	82% (137)	58% (7)	84% (130)	
Yes	16% (43)	18% (30)	42% (5)	16% (25)	
Tobacco use					0.01
No	72% (188)	69% (115)	33% (4)	71% (111)	
Yes	28% (74)	31% (51)	67% (8)	28% (43)	

### Proportion who do not obtain abortions due to Medicaid not paying

5.3% of participants [95% CI 2.9, 8.7] reported Medicaid not paying for abortion as a reason for not having an abortion. As a validity check, using the secondary indicator of women whose open-ended responses mentioned funding, this would be 4.1% [95% CI 2.1, 7.2]. Among women with Medicaid insurance (*n* = 167), 7.2%, [95% CI 4.1, 12.3] reported Medicaid not paying as a reason.

As validity checks, among women with Medicaid insurance, 92% reporting Medicaid not paying as a reason for not having an abortion preferred abortion at pregnancy discovery, compared to 10% who did not report this reason. 17% of those reporting Medicaid not paying as a reason preferred abortion at prenatal care entry, compared to 1% who did not report this reason. 58% of those reporting Medicaid as a reason for not having an abortion had unplanned pregnancies, compared to 11% unplanned pregnancies among those who did not report this as a reason. 17% of those reporting Medicaid not paying as a reason reported low certainty about their pregnancy outcome decision, compared to 5% not reporting this reason. [See Table 2].

**Table 2:** Validity checks

	Total	Medicaid population	Medicaid as a reason (Medicaid population denominator)		*P*-value among Medicaid population
N	265	167	12	155	
			Yes	No	
Pregnancy intentions (lmup)					< 0.001
Unplanned	12% (31)	15% (24)	58% (7)	11% (17)	
Ambivalent	64% (168)	63% (105)	33% (4)	66% (101)	
Planned	25% (65)	22% (37)	8% (1)	23% (36)	
Decisional certainty					0.001
High certainty	77% (196)	78% (129)	33% (4)	82% (125)	
Medium certainty	19% (48)	16% (26)	50% (6)	13% (20)	
Low certainty	5% (12)	6% (10)	17% (2)	5% (8)	
Preferred abortion at pregnancy discovery					< 0.001
No	86% (191)	84% (141)	8% (1)	90% (140)	
Yes	14% (74)	16% (26)	92% (11)	10% (15)	
Prefer abortion now					0.03
No	98% (228)	98% (162)	83% (10)	99% (152)	
Yes	2% (37)	2% (4)	17% (2)	1% (2)	

Three participants who reported Medicaid as a reason reported that they were still considering abortion for this pregnancy. All three of these participants were in the first trimester. In addition, most participants who reported Medicaid as a reason also reported a policy-related reason in response to the open-ended questions. Among those with Medicaid insurance, 4.2% reported both Medicaid as a reason in response to the direct question and a policy-related reason in the open-ended questions.

**Fig. 1:**
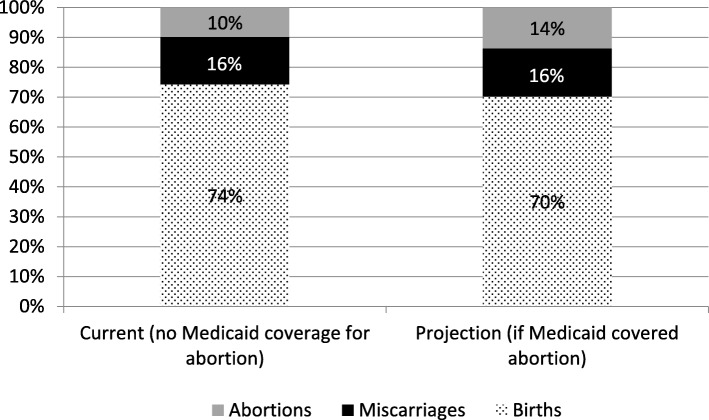
Estimated Pregnancy Outcomes in Louisiana

Based on published numbers, approximately 10% of pregnancies in Louisiana end in abortion. If Medicaid paid for abortion, this would increase to 14% [95% CI 12, 16]. [See Fig. 1] This means about 29% [95% CI 19, 41] of Medicaid-eligible pregnant women who would have an abortion if Medicaid covered abortion instead give birth. Applying 7.2% to the number of Medicaid births in Louisiana in 2015 (41,931), approximately 3000 [95% CI 1700, 5200] Louisiana women with Medicaid give birth per year instead of having an abortion because Medicaid does not cover abortion.

#### Characteristics of those who do not obtain abortions due to Medicaid not paying

Among women with Medicaid insurance, age, race/ethnicity, parity, and most measures of socioeconomic status were not associated with reporting Medicaid as a reason for not having an abortion. More women who reported Medicaid as a reason were not in a romantic relationship with the man involved in the pregnancy (58% v. 18%), had less than high school education (42% v. 22%), had alcohol use disorder risk (75% v. 21%), used drugs (42% v. 16%), and used tobacco (67% v. 28%). [See Table 1].

## Discussion

A previous systematic review estimated that about one-fourth of Medicaid-eligible pregnant women give birth instead of having an abortion due to Medicaid not covering abortion [9]. Using a different methodological approach, we arrived at an estimate for Louisiana substantially similar to the overall estimate from a decade-old systematic literature review [9]. The earlier estimate was based on literature published primarily the 1980s through early 2000s and which used primarily econometric methods and included multiple studies across multiple geographies [9]. This suggests pregnant women may accurately report both about considering abortion and Medicaid as a barrier to abortion care; this is consistent with research findings that women’s impressions of abortion costs and Medicaid coverage for abortion are generally accurate [15].

Recent research has paid considerable attention to how laws that seek to dissuade women from having abortions (such as waiting period and ultrasound laws) affect women’s ability to obtain and experiences obtaining abortions [29, 30]. This research has found that these laws do little to change women’s minds, but do increase financial costs, have emotional and social costs, and lead to care delays [29]. While recent Medicaid funding restrictions research has documented financial and emotional hardships associated with having to raise money to pay for abortion [7, 12, 14, 15], it has not focused on Medicaid restrictions as a barrier to obtaining an abortion. This study confirms that Medicaid funding restrictions for abortion continue to function as an insurmountable barrier to obtaining an abortion, specifically for women in Louisiana.

We also note that women with Medicaid insurance with alcohol use disorder risk, who used drugs, and who used tobacco were more likely to report lack of Medicaid coverage as a reason for not having an abortion. It is unclear whether this is due to being more likely to consider abortion or more likely to have difficulty overcoming funding barriers to obtaining abortions. Other research indicates that being unable to obtain an abortion is unlikely to contribute to sustained reduction in problematic alcohol use or in drug or tobacco use during pregnancy or the postpartum period [31].

There are several assumptions in this analysis that could affect the accuracy of these findings. However, examining these assumptions does not indicate that the estimate of the proportion of pregnancies among low-income women that end in birth rather than abortion when Medicaid does not pay is likely to be outside of our 95% CI. Specifically, the main question asked whether lack of Medicaid funding was a reason for having an abortion, not the only reason. Some participants who reported Medicaid as a reason also reported personal or interpersonal reasons for not having an abortion. However, even if we restrict the Medicaid as a reason proportion to those who reported a policy-related reason in response to open-ended questions, the estimate is 4.2%, within the 95% CI of our estimate. Similarly, three participants who reported Medicaid as a reason were still considering abortion upon prenatal care entry. Even if all three proceeded to have an abortion, this would still be within the 95% CI of our estimate. The sample excludes women who did not receive prenatal care. Nationally, about 1.4% of women do not receive any prenatal care [32]. Even if all the women who did not receive prenatal care would have had an abortion had Medicaid paid, this would increase our estimate of those who reported Medicaid as a reason to 8.6%, which is still within the upper limit of our 95% CI for this estimate.

This study has limitations. First, this study was conducted at three prenatal clinics in one region of one state. Findings may not be generalizable to other states with different demographics, different numbers of abortion providers, different local abortion fund practices, and different overall policy climate. Second, estimates are based, in part, on self-report data about considering abortion during pregnancy and reasons for not having an abortion. To check for possible underreporting from self-report data, we performed validity checks using data from open-ended responses and checking whether our outcome was associated with expected predictor variables, pregnancy intentions, and decisional conflict. Third, the association between substance use and reporting Medicaid as a reason could be due to self-report bias [33], with women more willing to report one also more willing to report the other. However, pregnant women who use alcohol and drugs face considerable barriers to prenatal care [34]. They may face similar barriers to abortion. Fourth, our estimates are likely imprecise. We have a somewhat wide confidence interval for reporting Medicaid as a reason for not having an abortion. However, our confidence interval overlaps with confidence intervals from the decade old systematic review [9] estimate, suggesting plausible accuracy.

This study also has strengths. First, we had high participation (86%). Second, we used an innovative approach to derive an up-to-date estimate of the impact of lack of Medicaid funding for abortion. This approach yields a finding consistent with previous estimates, suggesting the previous estimate is still valid.

## Conclusions

Forty years after the Hyde Amendment was passed, lack of Medicaid funding for abortion continues to have substantial impacts on women’s ability to obtain abortions in Louisiana.

## Data Availability

The datasets generated and/or analyzed during the current study are not publicly available due to the terms of participant consent but are available from the corresponding author on reasonable request.

## Abbreviations

GED: General Education Diploma
HS: High School
LMUP: London Measure of Unplanned Pregnancy
